# RETRACTED: Tasca, F.; Antiochia, R. Biocide Activity of Green Quercetin-Mediated Synthesized Silver Nanoparticles. *Nanomaterials* 2020, *10*, 909

**DOI:** 10.3390/nano16160971

**Published:** 2026-08-07

**Authors:** Federico Tasca, Riccarda Antiochia

**Affiliations:** 1 Departamento de Química de los Materiales, Facultad de Química y Biología, Universidad de Santiago de Chile, Av. Libertador Bernardo O´Higgins 3363, Santiago 9170022, Chile; federico.tasca@usach.cl; 2 Department of Chemistry and Drug Technologies, Sapienza University of Rome, P.zale Aldo Moro 5, 00185 Rome, Italy

The journal retracts the article “Biocide Activity of Green Quercetin-Mediated Synthesized Silver Nanoparticles” [1] cited above.

Following publication, concerns were raised regarding substantial overlap between this article [1] and a previously published paper by several of the same authors [2], as well as the potential unauthorized reuse of copyrighted material.

In accordance with standard journal procedures, the Editorial Office and Editorial Board conducted an investigation. The investigation confirmed substantial, unacknowledged overlap between the two publications [1,2], including figures and methodological content, and identified seven images that had been reproduced without appropriate permission. As a result, the Editorial Board determined that the article does not meet the journal’s standards for publication integrity and has decided to retract it in accordance with MDPI’s retraction policy.

This retraction was approved by the Editor-in-Chief of *Nanomaterials*.

The authors have been informed of this retraction and have indicated their disagreement with this decision.

## References

[1] Tasca F., Antiochia R. (2020). RETRACTED: Biocide Activity of Green Quercetin-Mediated Synthesized Silver Nanoparticles. Nanomaterials.

[2] Bollella P., Schulz C., Favero G., Mazzei F., Ludwig R., Gorton L., Antiochia R. (2017). Green synthesis and characterization of gold and silver nanoparticles and their application for development of a third generation lactose biosensor. Electroanalysis.

